# Die Krankheiten des Verdauungs Kanals (Œsophagus, Magen, Darm), Ein Leitfaden für Praktische Ärzte

**Published:** 1905-09

**Authors:** 


**Die Krankheiten des Verdauungs Kanals (Œsophagus, Magen, Darm), Ein Leitfaden für Praktische Ärzte.** Von Dr. PAUL COHNHEIM. Pp. 247. Berlin: S. Kaiger. 1905.

The object of this book is to provide the general practitioner with a short manual of diseases of the alimentary tract, and to give him an account of the newer methods of investigation and treatment. At the same time the author has restricted himself to the descriptions of those methods of diagnosis which do not require any very elaborate apparatus or the use of an extensively-equipped laboratory, but can at once and readily be carried out in the routine of daily practice. His aim is to describe methods simple in themselves, but by whose means, if properly applied, a complete and accurate diagnosis can be arrived at. The author has worked for many years in Dr. Boas's (of Berlin) clinic, and has therefore had a wide experience.

He lays stress upon subjective symptoms, pointing out that by the history of the illness and a careful analysis of symptoms one may often proceed far towards the diagnosis. There is no doubt that the tendency has been of late, from the importance attached to the chemical investigation of the gastric contents, to pay insufficient attention to symptoms in gastric disorders, and it may further be remarked that these chemical studies have enabled us, after carefully correlating their results with symptoms, to make a more accurate use of the latter. The author attaches considerable importance in the diagnosis of functional from organic diseases of the stomach to the absence of severe pain (gastralgia) in the former.

There is a good section on the subject of erosions and fissures in the pyloric region of the stomach which covers somewhat new ground, as it is the outcome of recent observations. The author does not admit that hyperchlorhydria and hypersecretion are primary causes of pyloric spasm, which again produces gastric dilatation; but on the contrary holds that there is always some pyloric lesion first, and that these other phenomena are secondary to it.

There is a good section on Cancer of the Stomach, and, speaking generally, we consider the first part of the book, which deals with diseases of the stomach, better than the second, which is concerned with those of the intestines. This is partly due to the fact that the second part is much shorter, and therefore the subject is necessarily treated briefly. We should allude, however, to the really excellent chapter on Constipation, in which the author gives a good practical division of the different stages in the course of chronic constipation and much useful advice as to treatment.

A short section on Diseases of the Rectum, diet-tablets for the different disorders treated of in the whole book, and epitomes of methods of treatment and of symptoms complete the work, which has also an index. There are a few illustrations of the microscopic appearances of the deposits from the gastric contents and of the fæces.

In conclusion, this seems to us an excellent manual, and that the author has succeeded in his task. The section on Diseases of the Stomach is especially good. The book is thoroughly practical, clearly written, and the advice as to treatment marked by sound sense and wide knowledge. Full and careful directions are given as to the treatment of each disease. The advice as to diet is of course adapted to German habits of life, but any intelligent reader can obtain the necessary rules from it. We consider this an excellent book for any practitioner who reads German, and one from which he can derive considerable help in his daily work.

